# IL-18 Signaling Is Essential for Causing Streptococcal Toxic Shock-like Syndrome (STSLS)

**DOI:** 10.3390/life12091324

**Published:** 2022-08-26

**Authors:** Lei Xu, Yue Zeng, Peiying Gao, Xi Lu, Kunlong Xia, Liting Zhou, Chengfeng Zhang, Chenyang Yi, Anding Zhang

**Affiliations:** 1 State Key Laboratory of Agricultural Microbiology, Hubei Hongshan Laboratory, College of Veterinary Medicine, Huazhong Agricultural University, Wuhan 430070, China; 2 Key Laboratory of Preventive Veterinary Medicine in Hubei Province, The Cooperative Innovation Center for Sustainable Pig Production, Wuhan 430070, China; 3 Key Laboratory of Development of Veterinary Diagnostic Products, Ministry of Agriculture of the People’s Republic of China, Wuhan 430070, China; 4 International Research Center for Animal Disease, Ministry of Science and Technology of the People’s Republic of China, Wuhan 430070, China

**Keywords:** *Streptococcus suis* serotype 2, streptococcal toxic shock-like syndrome, interleukin-18, NLRP3 inflammasome

## Abstract

*Streptococcus suis* (*S. suis*) is an emerging zoonotic pathogen that can cause multiple diseases, including streptococcal toxic shock-like syndrome (STSLS). The *S. suis* SC-19 strain could cause NOD-like receptor thermal protein domain-associated protein 3 (NLRP3) inflammasome hyperactivation, then induce a cytokine storm and STSLS. Although IL-18 is the downstream effector of NLRP3 signaling, the role of IL-18 signaling on STSLS remains to be elucidated. Thus, *il18r1* gene knockout mice were constructed and challenged with the SC-19 strain. Alleviated clinical signs and tissue damages, as well as improved survival were observed in *il18r−/−* mice compared with the WT mice post-SC-19 challenge. Meanwhile, an obvious decrease in the inflammatory cytokine levels in blood was observed in the *il18r-/-* mice infected with SC-19. Therefore, IL-18, the downstream effector of NLRP3 inflammasome activation, was responsible for the cytokine storm and STSLS development caused by *S. suis*, suggesting that IL-18/IL-18Rα signaling could serve as a new target for STSLS.

## 1. Introduction

*Streptococcus suis* (*S. suis*) is a common and important zoonotic agent that can cause huge economic losses in the swine industry of the world and lead to a significant threat to the public health of humans and immunocompetent patients [1,2]. *S. suis* infection in humans causes a variety of serious diseases, including meningitis, endocarditis, arthritis, and sepsis [3]. In 1968, human infection with *S. suis* was first reported and, as of now, more than 1600 people have been infected with *S. suis*, primarily in Asia, including China, Vietnam, and Thailand [3,4,5,6]. However, in 1998 and 2005, the outbreak of *S. suis* serotype 2 unusually caused two large-scale human *S. suis* epidemics, in which 240 humans were infected and 53 died in China [7,8,9]. Moreover, 97.4% of fatal cases were observed with streptococcal toxic-shock-like syndrome (STSLS), including the hallmarks of acute high fever, blood spots, shock, vascular collapse, hypotension, dysfunction of multiple organs, and acute death [8,10], which has attracted widespread attention of the world.

The clinical investigations showed that STSLS patients died with severe inflammation, characterized with cytokine storms and dysfunction of multiple organs [7]. Subsequent studies further confirmed that the production of inflammatory cytokine storms was critical for STSLS [11], and inhibiting excessive inflammatory response could significantly alleviate acute death caused by STSLS [12,13]. Our previous study indicated that high expression of suilysin (SLY) was required for the highly pathogenic *S. suis* strain SC-19 to cause NOD-like receptor thermal protein domain-associated protein 3 (NLRP3) inflammasome hyperactivation, which in turn causes the induction of cytokine storms and STSLS [14]. This was also confirmed by another group [15]. However, it remains to be elucidated how NLRP3 activation causes severe inflammation. 

The activation of inflammasomes could lead to pro-caspase-1 into an active protease, which mediates the processing of several targets: it cleaves pro-IL-1β and pro-IL-18 into their biologically active forms and also cleaves gasdermin D (GSDMD), which leads to a particular form of cell death called pyroptosis for secretion of mature IL-1β and IL-18 [16,17,18,19,20]. IL-1β is required for controlling bacterial burdens caused by the ST1 strain but not by the ST7 strain, and *il1β* gene-deficient mice were more susceptible to *S. suis* strains in [21]. This was in coincidence with the results of an earlier study indicating the protective role of IL-1 on the resistance to *S. suis* infection [22]. However, GSDMD-deficient mice were also resistant to STSLS development, indicating that pyroptosis, rather than IL-1β signaling trigged by inflammasome activation, promotes STSLS development [15]. IFN-γ, induced uniquely by IL-18 but not by IL-1β [23,24], also played a broad and important role in severe inflammatory responses and organ injury during shock syndrome [11,25,26]. Furthermore, the highly virulent strain SC-19 caused higher levels of IL-18 and IFN-γ than the meningitic strain P1/7 [27]. These authors suggested that IL-18, as a NLRP3 downstream signaling molecule, may induce high levels of IFN-γ during STSLS. However, the role of IL-18 on STSLS remains to be elucidated. 

Therefore, the study aimed to clarify the role of IL-18 signaling during the development of STSLS, and also to delineate signaling cascades for severe inflammatory response during STSLS.

## 2. Materials and Methods

### 2.1. S. suis Strain and Culture Conditions

The *S. suis* serotype 2 epidemic strain SC-19 (ST7) was used in the present study. The epidemic strain SC-19 was originally isolated from the brain of a diseased pig in China and has been widely used in several models of STSLS [28,29]. *S. suis* was cultured in tryptic soy broth (TSB, Difco Laboratories, Detroit, MI, USA) or on tryptic soy agar (TSA, Difco Laboratories, Detroit, MI, USA) plates with 10% (vol/vol) newborn bovine serum (Sijiqing, Hangzhou, China) at 37 °C. 

### 2.2. Ethics Statement 

The mice used in this study were female, 4 to 6 weeks old C57BL/6, and were bred and housed in SPF conditions. All experiments involving infectious *S. suis* were conducted in strict accordance with the Guide for the Care and Use of Laboratory Animals Monitoring Committee of Hubei Province, China, and approved by the Scientific Ethics Committee of Huazhong Agricultural University. All efforts were made to minimize the suffering of the animals used in the study.

### 2.3. Construction of il18r1 Gene Knockout Mice

In order to evaluate the role of IL-18 on STSLS, *interleukin-18 receptor 1 (il18r1)* gene knockout mice were constructed using the CRISPR/Cas9 gene-editing system as previously described [30,31]. In brief, zygotes were collected from sexually immature female C57BL/6 mice. Then, an sgRNA (5′-GCCACCATGAGATGGTTCAA-3′) targeting exon 4 of the *il18r1* gene (MGI: 105383) and the Cas9 mRNA were injected into the cytoplasm of pronuclear stage embryos. Finally, the injected embryos were transferred into the oviduct of the recipient mother for KO mouse productions. The newborn mice were genotyped by PCR followed by DNA sequencing analysis. The wild-type, heterozygote, and knockout mice were used for control with each other. Primers for genotyping were as follows: il18r-F, 5′-AGGGGGATCAGGGAAAAATCAC-3′ (forward); il18r-R, 5′-AAATTTAGAGTGACTGGCTTAATA-3′ (reverse); il18r-f1(wt), 5′-CACCATGAGATGGTTCAAAGG-3′ (forward); and il18r-f2(ko), 5′-CACCATGAGATGGTTGTGCTT-3′ (forward). The DNA fragment for WT and HET was 405 bp, and the DNA fragment for KO was 390 bp. All mice were bred and housed in SPF conditions. 

### 2.4. Experimental Infections of Mice

Female, four- to six-week old *il18r1* gene knockout mice (*il18r1-/-*) and C57BL/6 (*il18r1+/+*) mice (10 mice per group) were challenged with 4 × 10^8^ CFUs of SC-19 by an intraperitoneal (i.p.) injection to directly evaluate the effect of IL-18 on STSLS development [27]. The clinical scores were assigned based on depression, swollen eyes, rough hair coat, and lethargy. The details were described as follows: 0 =  normal response to external stimuli; 1 = ruffled coat and slow response to external stimuli; 2 =  responds only to repeated stimuli; 3 = no response to external stimuli or walking in circles; and 4 = dead. Mice exhibiting extreme lethargy or neurological signs (score =  3) were considered moribund and were humanely euthanized [32].

To further analyze the role of IL-18 on STSLS, the level of cytokines and bacterial burden were also evaluated during *S. suis* infection [31]. At 6 h and 12 h post-infection, mice were euthanized, bacterial loads were measured in fifty microliters of blood and the remaining blood was used to analyze the level of cytokines. Half of the liver, lung, tissues, and brain were used for bacterial load analysis. The remaining lung, liver, and spleen tissues were fixed in 10% neutral buffered formalin for histopathology examinations.

### 2.5. Multiplex Cytokine Assays

Serum was obtained by centrifugation at 500 g for 30 min at 4 °C, and stored at −80 °C until analysis. Serum levels of cytokines TNF-α, IFN-γ, IL-6, IL-1β, IL-17A, and IL-12p70 were measured in all mice samples, and quantified using U-PLEX electrochemiluminescence ELISA (Meso Scale Discovery, MD, USA). Data acquisition was achieved by MESO^TM^ QuickPlex SQ120 (Meso Scale Discovery, MD, USA) and analyzed in the MSD Discovery Workbench Desktop 4.0 software (Meso Scale Discovery, MD, USA), where values were expressed in pg/mL for each cytokine.

### 2.6. Histopathology Examinations 

Tissues were fixed in 10% neutral buffered formalin for over 24 h. After embedding in paraffin, tissues were cut into 2–3 μm sections. Subsequently, dewaxing was performed through a xylene and ethanol series to deionized water. Finally, the sections were stained with hematoxylin and eosin (H&E) following standard procedures [13], and examined under light microscopy (Olympus, Tokyo, Japan).

### 2.7. Bacterial Load in The Blood and Tissues

The colonization capabilities of the SC-19 strain were detected in *il18r1-/-* mice and *il18r1+/+* mice as described previously [33]. Briefly, bacterial counts in blood were determined by plating serial dilutions on TSA plates. The tissues were weighed, homogenized, serially diluted, and then plated on TSA plates to evaluate the bacterial counts [34].

### 2.8. Statistical Analysis

GraphPad Prism 6 software was used in data analysis by two-tailed, unpaired *t*-tests. A log-rank test and two-way RM ANOVA were used to compare survival rates and clinical scores, respectively. All assays were repeated ≥3 times. For all tests, a value of *p* < 0.05 was considered to be of significance.

## 3. Results and Discussion

### 3.1. Construction of il18r1 Gene Knockout Mice

**Figure 1:**
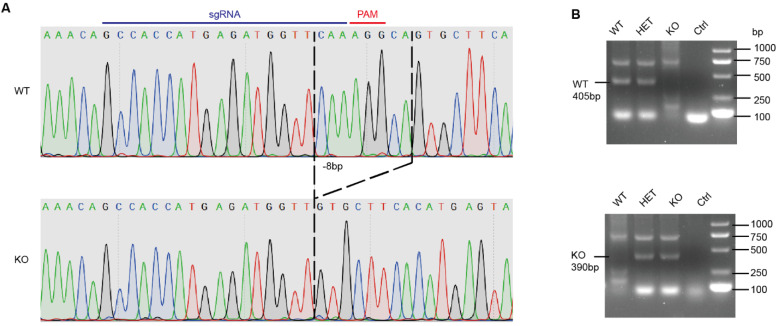
Confirmation of *il18r1-/-* mice. (**A**) DNA sequencing of the *il18r1* gene in the *il18r1-/-* and *il18r1+/+* mice. An 8bp DNA sequence was deleted in the *il18r1-/-* mice. (**B**) Genotyping by PCR. A 390 bp DNA fragment for KO and HET. A 405 bp DNA fragment for WT and HET. WT: wild-type mouse; HET: heterozygote mouse; KO: knockout mouse. Ctrl: negative control (H2O).

In order to analyze the effects of IL-18 on STSLS, *il18r1* knockout mice were constructed with the CRISPR/Cas9 system (Figure 1). Sanger sequencing indicated that there is 8 bp deletion (CAAAGGCA) in the fourth exon of the *il18r1* gene in the *il18r1–/–* mouse. It indicated the frameshift of the *il18r1* gene, suggesting successfully constructed *il18r1* gene knockout mice.

### 3.2. Knockout of il18r1 Could Significantly Decrease Mortality during STSLS

**Figure 2:**
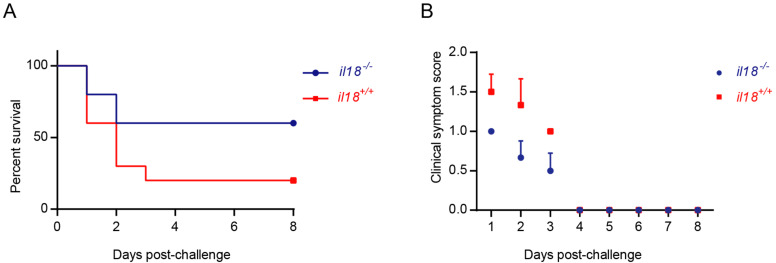
Knockout of *il18r1* could decrease mortality during STSLS. The wild-type mice (*il18r1+/+*) and the *il18r1*-deficient mice (*il18r1-/-*) were infected (i.p.) with SC-19. (**A**) Survival of infected mice (*n* = 10). (**B**) Clinical symptom scores of infected mice; deceased mice were excluded (n = 10). *il18r1+/+*: wild type mouse. *il18r1-/-: il18r1* knockout mouse.

Highly virulent *S. suis* infection induces STSLS, which is characterized by inflammatory cytokine storms, multi-organ damages, and ultimately, acute death [7,8]. In order to further analyze the function of IL-18 on STSLS, *il18r1-/-* and *il18r1+/+* mice were challenged with the highly virulent *S. suis* strain SC-19. As described before, the SC-19 strain caused severe death in *il18r1+/+* mice [34]. However, morbidity and mortality caused by *S. suis* infection were significantly decreased in *il18r1-/-* mice, and acute death was also reduced (Figure 2). This indicated that IL-18 might play a vital role on STSLS caused by the highly virulent strain SC-19. 

### 3.3. Knockout of il18r1 Could Decrease the Tissue Damages during STSLS 

**Figure 3:**
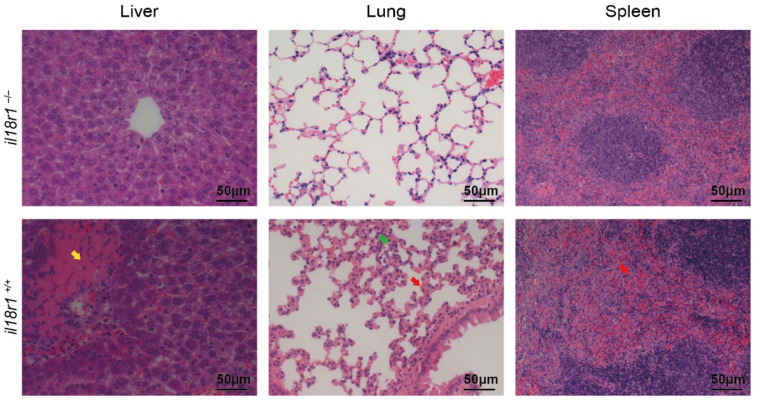
Knockout of *il18r1* could alleviate the tissue damages during STSLS. H&E staining of tissue sections of mice at 6 h post-infection infected with *S. suis*. Necrosis in the liver: yellow arrow; congestion in the lung and spleen: red arrow; infiltration of inflammatory cells in the lung: green arrow. Scale bar indicates 50 μM. *il18r1+/+*: wild-type mouse group. *il18r1–/–: il18r1* knockout mouse group.

As described before [14], infection with the SC-19 strain can cause acute multi-organ dysfunctions in the *il18r1+/+* mice, such as severe congestion and infiltration of inflammatory cells in the lung, necrosis and vacuolated degeneration in the liver, and congestion in the spleen (Figure 3). However, the infection on the *il18r1-/-* mice did not show severe tissue injury. The results indicated that IL-18 might play an essential role in multi-organ dysfunctions and acute death during STSLS.

### 3.4. Knockout of il18r1 Decreases Serum Inflammatory Cytokine Storms but Does Not Significantly Decrease Bacterial Load

**Figure 4:**
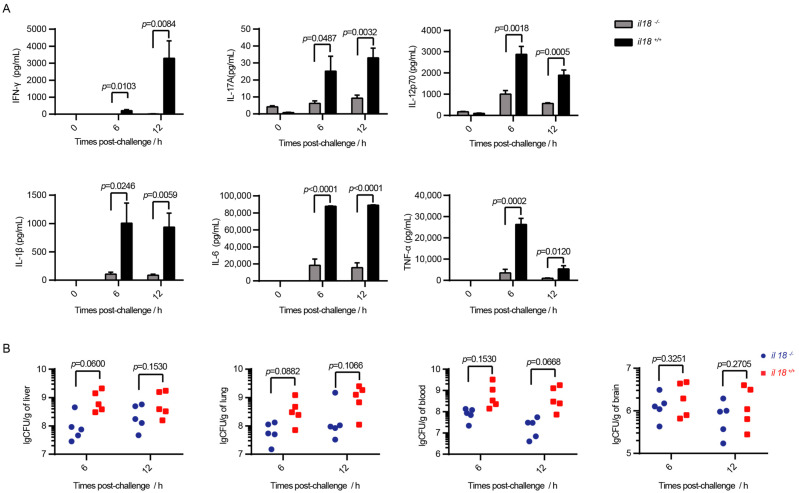
Knockout of il18r1 decreases serum inflammatory cytokine storm. The wild-type mice (*il18r1+/+*) and the *il18r1*-deficient mice (*il18r1-/-*) were infected (i.p.) with *S. suis* SC-19. (**A**) Cytokine levels in the blood at 6 h and 12 h post-infection were determined (*n* = 5). (**B**) The bacterial burdens in the liver, lung, blood, and brain at 6 h and 12 h post-infection were determined (*n* = 5). Each symbol represents the bacteria recovered from 1 mouse. Error bars represent the mean ± standard deviations. *il18r1+/+*: wild-type mouse group. *il18r1-/-: il18r1* knockout mouse group.

The SC-19 strain can cause high levels of inflammatory cytokine storms and high bacterial burden in mice [14]. Although IL-18 signaling was reported to play a role in bacterial control and protection against *Streptococcus pneumoniae* and *Streptococcus agalactiae* infection [35,36,37,38], there was no significant difference in bacterial load of various tissues in *il18r-/-* mice and *il18r1+/+* mice (Figure 4). This indicates different roles of IL-18 in different bacterial infections.

However, the levels of blood cytokines such as IL-17A and IFN-γ were significantly reduced in *il18r-/-* mice compared with *il18r1+/+* mice (Figure 4). This indicates that IL-18, one of the downstream effectors for NLRP3 inflammasome activation, was responsible for inflammatory cytokine storms and STSLS. In addition, the Chinese epidemic *S. suis* strain induced a strong and fast IFN-γ response by NK cells. Furthermore, IFN-γ-deficient mice infected with the epidemic *S. suis* showed significantly better survival rates than wild-type mice [11]. The decreased level of IFN-γ in *il18r-/-* demonstrates that IL-18 was responsible for IFN-γ induction during STSLS development. This suggests that NLRP3/IL-18/IFN-γ signaling was responsible for the STSLS development, which could serve as a new target for STSLS.

### 3.5. Limitation

The CRISPR/Cas9 system has become the mainstream technology for gene knockout, even if it has off-target potential. To illustrate the function of IL-18, the *il18r1* gene knockout mice were constructed based on this technology, so we could not completely rule out the side effects due to off-target potential.

## 4. Conclusions

**Figure 5:**
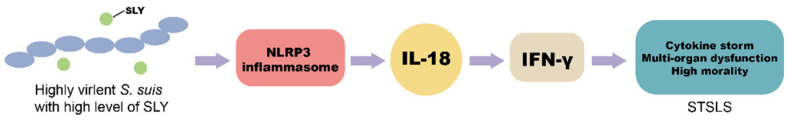
Scheme of the relationship of STSLS and IL-18. Previous study has illustrated that SLY was the essential and sufficient condition for NLRP3 inflammasome hyperactivation, causing cytokine storms and STSLS [14]. IFN-γ has been demonstrated to activate cytokine storms and STSLS [11]. Our study showed that IL-18, the downstream of NLPR3 signaling, was responsible for IFN-γ induction and STSLS development.

A previous study has demonstrated that SLY was the essential and sufficient condition for NLRP3 inflammasome hyperactivation, causing cytokine storms and STSLS [14]. The present study indicated that IL-18, the downstream of NLPR3 signaling, was responsible for IFN-γ induction and STSLS development (Figure 5).

## Data Availability

All data are available within the article or from the corresponding author upon reasonable request.
